# “Self-development matters” - Perception of Sakhis (CHWs) assessing self-development outcomes of their participation in the HBNC Program

**DOI:** 10.1186/s12905-018-0536-x

**Published:** 2018-02-21

**Authors:** Anagha Anand Mahajani, Abhijit Shrinivas Prabhughate, Pearl Tiwari, Shubhangi Sohoni, Ajay Gajanan Phatak, Vallaree Anant Morgaonkar, Somashekhar Marutirao Nimbalkar

**Affiliations:** 1 Program Monitoring and Research, Ambuja Cement Foundation, Mumbai, 400059 India; 2 Central Research Services, Charutar Arogya Mandal, Karamsad, 388325 India; 3 Department of Paediatrics, Pramukhswami Medical College, Karamsad, 388325 India

**Keywords:** Newborn care, Self-esteem, Work, Community health workers

## Abstract

**Background:**

Community Health Workers (CHWs) play an instrumental role in promoting socio-behavioural change at the community level, which results in changed indicators of community health. While outcomes are mostly reviewed for achieving program objectives, it is pertinent to understand the process of program implementation mainly from the perception of participating CHWs.

**Methods:**

A qualitative study to understand the perception of Sakhi’s (CHWs) regarding the outcomes of their participation in Home-Based Neonatal Care (HBNC) Program implemented by a non-governmental organization (NGO). Data consisted of 3 FGDs and 20 in-depth unstructured interviews with participating Sakhis.

**Results:**

Sakhis perceived their ability to take decisions at critical phases of the program as an important factor influencing their performance. The opportunity to participate as a Sakhi in the health programme initiated a process of change at the personal level. The changes perceived by Sakhis were enhancement in knowledge, skills and capabilities of Sakhis. The combination of improved skills, knowledge and attitude had culminated in the process of experiencing self-empowerment for the participating Sakhis. Their ability to positively influence the individuals and community with their initiatives to improve women and child health and save lives in critical situations facilitated development of a new identity and improved societal status in their communities. Changed power-relations at the family and community level promoted the involvement of Sakhis in the broader development agenda. Sakhis’ ability to strategize goals, evaluate their own abilities, their willingness to upgrade knowledge and take others along in bringing social change, was an evident movement towards self-development.

**Conclusion:**

An opportunity for local women to participate in development programs creates potential for self-development as a cascading effect in addition to the accomplishment of planned program objective.

## Background

The use of community health workers (CHWs) has for long been one of the important strategies to address the issue of healthcare outreach. India has a rich history of engaging CHW in health prevention and promotion programmes. A large national CHW scheme was established in the late 1970s that aimed to provide one CHW for every 1000 people in order to provide adequate health care to rural people and to educate them in matters of preventive and promotive health care [1]. This approach of involving CHW for larger health and development goals continued in India and also got a fillip with the Alma Ata declaration of 1978, which called for integrated approaches to healthcare in low-income countries [2], and the Cairo Population Conference in 1994, which recognized the need for involvement of communities for providing sexual and reproductive health services [3]. Thus, over the three decades (1970s – 2000), the concept of CHW gained currency and CHWs came to be recognised as an effective cadre of local men and women chosen by the community, trained to deal with health problems of individuals and community, and to work in close relationship with the government services. In 2005, India embarked on a massive reform of its health system by forming the National Rural Health Mission (NRHM), under which it legitimized the role of CHW by forming a cadre of women CHW called Accredited Social Health Activists (ASHA) [4]. Presently the ASHA play a pivotal role in providing health care services in remote rural geographies of India.

Being firmly rooted in communities, the CHW play an instrumental role in promoting socio-behavioural change at the community level, which is expected to result in improvements in individual and community’s health indicators. While several studies [5–8] have sought to understand the role of CHWs as well as their effectiveness in improving health scenario at the community level, not much is known about the effect of a CHW’s work on her individual self. This aspect deserves to be understood because CHWs in India are typically women from villages who, prior to becoming a CHW, are unexposed to the development process and after entering this new domain of work often continue to operate in an unfavourable or hostile environment. We present in this paper Sakhis’ (meaning female friend in vernacular language) perception of how being a CHW in a home-based neonatal care (HBNC) program impacted their selves. The Sakhis shared their experiences while explaining about the challenges faced by them and the mitigation strategies they adopted.

### Sakhi’s work in home-based neonatal care Programme

Started in 2005, a HBNC program was implemented for over a decade in 160 villages of three blocks in Chandrapur District, Maharashtra by Ambuja Cement Foundation, the corporate social responsibility (CSR) arm of Ambuja Cements Ltd. In this program women from villages who wanted to become CHW were selected and trained to provide services in communities on the lines of the well-known HBNC model proposed by Bang et al. [9] The Sakhis were responsible for generating awareness on health issues, providing ante- and post-natal care services, promoting institutional deliveries and monitoring the overall health of mother and child. They had to develop strong bonds with women, in-laws and family members, community members, and health providers so that they could effectively deliver their responsibilities.

### Literature review

#### Who are community health workers?

The umbrella term “community health worker” (CHW) embraces a variety of community health aides selected, trained and working in the communities from which they come. The CHW’s roles and activities are tailored to meet the unique needs of their communities. It also depends upon whether they work in the healthcare or social services sector [10]. Although CHWs can be men or women, young or old, literate or illiterate, the available evidence shows young and middle aged women prominently playing the role of CHWs. The CHW are grass-root voluntary workers who work in the same community in which they reside. This feature helps them to identify the problems of the community people in a better way and to find solutions for these problems through community involvement and participation.

#### Self-development and empowerment of community health workers

Self-development and empowerment are inherently interrelated. In the context of health and social development, the concept of “empowerment” has received much more attention. Empowerment, both as a process and a goal, has taken considerable prominence within community organisation theories, especially as operationalized by NGOs since 1970s [10]. It has been defined as “a group’s or individual’s capacity to make purposive choices, that is, to make choices and then to transform those choices into desired action and outcome’” [11]. Women’s empowerment, introduced as a concept at the International Women Conference in 1985 at Nairobi, was defined as redistribution of social power and control of resources in favour of women. Other definitions [12], such as one given by International Fund for Agricultural Development (IFAD) (1978–98) [13] have added that the poor men and women have limited or no access to resources, and socio-economic structures play a critical role in reducing or inhibiting access of women. Cultural traditions also inhibit women’s access to other productive resources and services. There has been almost no research that has explored the process of self-development or empowerment among CHW in India. Only one study explored the process of action on social determinants and it found that the CHWs developed identities as change agent and advocates for the community, both with respect to local culture and gender norms and in ensuring accountability of service providers [14]. One reason for the lack of past research could be that there are few CHW programmes that articulate and visualize this role for CHWs. Another factor may be that funding of CHW programmes and their evaluations are compelled to focus on individual health outcomes and thereby ignoring work on social determinants [1]. However, where these have been explicitly studied, it has been found that the CHWs can play the role of community advocates and “change agents, empowering individuals, their community, and themselves” [15]. There is hence a further need to explore outcomes of any development process involving women in a broader context. It is also important to study, besides the program aim, the perception of participating women about the outcome of their involvement in terms of individual development.

## Methods

The data presented in this paper are from an exploratory qualitative study that was conducted to understand the challenges faced and mitigation strategies used by Sakhis. The study was conducted adopting a grounded theory approach and data acquired was from 3 focus group discussions and 20 in-depth interviews with Sakhis who met the qualifying criterion of having at least 2 years of experience. A more detailed description of the study’s setting, conceptual framework, process of data analysis, and findings related to challenges and mitigation strategies are reported in another paper [16].

An unexpected recurrent theme emerged while analysing data. While narrating challenges and mitigation strategies many Sakhis had expressed about how they had undergone positive changes at a personal level. This theme was beyond the scope of conceptual framework, and therefore the authors revisited the data to develop a nuanced understanding of the Sakhi’s perception of the effect of her work on herself. Each interview was independently coded by at least two authors and the validity of coding was ensured through joint review of coded sections by three authors (AM, AP and SS). This helped to minimize bias of the authors and ascertained that findings were firmly rooted in experiences shared by the Sakhis. After engaging in several deep reflective discussions on the emerging theme, the authors concurred that the pattern reflected “self-empowerment” that had resulted from the process of working as a CHW.

## Results

Through the analysis of the data we reviewed the perception of Sakhis regarding the critical phases of their process of engagement and its outcome in terms of empowerment and self-development.

### Decision to participate as Sakhis

There were certain common characteristics observed among the local women willing to participate as Sakhis. Many of them were in the 20–35 year age-group, married, possessed minimal literacy levels, and in most cases their work was restricted to the household chores, though few were involved in farm labour to support the family livelihood.

In this phase the decision to participate was considered to be the most critical by the local women and the process of empowerment originated at this stage. Their decision was influenced by their family, chiefly by their spouses and in-laws. The Sakhis shared about the nature of support they experienced in joining the program. While some Sakhis experienced enthusiasm and support from their spouse and the immediate family, others did not. Despite lack of support many decided to become a Sakhi. The experiences shared by Sakhis showed a distinctive feature of women who had opted to become a Sakhi – a very strong desire to participate in activities beyond the realm of their household work and familial responsibilities. Though their motivation differed in degree, in most cases women regarded this as an opportunity to perform. High motivation and positive attitude influenced the ability of Sakhis to take a decision about participation. A Sakhi recalled,

*“I got married at the age of 15 and had a baby when I was 18. I was not allowed to talk to anybody. I hated to work in the farm and hence was annoyed with it but did not have any choice. It was then this opportunity for training came up. My in-laws and my husband opposed, but I argued with them to join this work. I left my six-month old baby to be taken care by my sister-in-law for five days during the first training. I suffered a lot because I missed my baby but didn’t quit the training”.*

For many Sakhis it was the first opportunity to independently express and assert their views. Once the Sakhis had asserted and decided to become a CHW, the decision itself helped Sakhis to further build their confidence to initiate work for the community’s health and development. For few Sakhis, an additional factor that helped move toward independent decision making was their selection by the community to become a CHW for their village. In certain cases however local women experienced dissuasion from the community. Their intention and interest in taking on the work as Sakhi were questioned and were mistrusted for their capability and eligibility to become a Sakhi.

ACF field supervisors also recommended some potential local women based on the previous interaction with the community. Sakhis perceived recommendation of their name as recognition of their efforts as well as their potential, which encouraged them to participate. A sense of self-esteem emerging out of recognition of their potential by the community enhanced the level of confidence of the women turned Sakhis. Initial fear was replaced with confidence to take upon challenges with the process of engagement. The Sakhis shared that gaining ability to respond to challenges was an elevating experience.

### Decision to act

The action phase began with the orientation training followed by field outreach. Each Sakhi developed a customised outreach plan and communication strategy for her village as part of the training. This helped Sakhis to have an improved acceptability for their health messages. Sakhis, being local and always available, got an opportunity to demonstrate their knowledge and skills. Their constant communication and follow-up helped in spreading health awareness especially in case of pregnancy. Sakhis experienced non-acceptance from the community during the initial period, however they continued their efforts to reach individuals and families with health care inputs and support especially during the critical conditions. In this regard, explaining how they were able to deal with difficulties, a Sakhi shared,

“*During the training we were prepared for this phase. We were told that the patience to take the community with you will count first followed by your ability to provide needed health care services*”.

Sakhis were able to adapt the style of communication through different phases of interaction with the community. They mentioned to have experienced improvement in communication skills, develop a poised attitude and ability to think creatively in a given context. The strong commitment and persistence among the Sakhis helped initiate the process of changing beliefs and practices among the community members. The process of influencing beliefs and attitudes of community also contributed to Sakhi’s improved confidence to act further.

### Intervening in difficult situations

The experiences shared by Sakhis indicated that the first experience of handling a critical case played a significant role in boosting their confidence. It was evident in the data that technical knowledge and timely decision making ability among the Sakhis got sharper with each critical case handled by them. The experience of handling critical child birth cases was perceived to be a significant differentiator by Sakhis. They placed highest value on handling critical cases that led to saving human life, considering it to be a noble task. Further, Sakhis mentioned it to be a gratifying experience that also elevated their status in the community. The ability to respond to critical cases, ability and courage to take timely decisions and recognition for this ability from their own community and other stakeholders developed an immense sense of self-esteem among the Sakhis. The aspects of saving lives of babies also provided Sakhis with huge satisfaction. As a Sakhi mentioned,

*“I can’t tell you how happy I feel to see the saved child grow and it is also the same with the family. I built relations with those families. The families have also shared with their child about their gratitude for the efforts we had made to save her”.*

Another Sakhi stated,

*“This is our reward. Money doesn’t matter to us but it is the respect that we get in the community which becomes important”.*

### Going beyond established roles

The Sakhis’ experiences revealed that their performance of roles was also shaped by their ability to influence social customs and practices. Acknowledgment by community members and health providers encouraged Sakhis to take initiative and also go beyond their predetermined role in the project and contribute in other aspects of community health and village development issues. Beginning with women and child care, they moved on to working on addressing issue of child marriage, sanitation, nutrition, decisions regarding contraception and quality of health care provided by public health system. Sakhis believed their effort helped in improving awareness among the community about relationship of health issues with social customs such as early age of marriage. Sakhis engaged with *panchayat* initially for health issues and expanded their reach to discussing other development schemes by government, being representative on various committees or to support social action campaign on issues such as alcoholism and tobacco use. Sakhis also helped women to get their ration card or access loan from a bank. These added roles further demanded mobility for a Sakhi. It was evident from the following example that the enhanced mobility and interface of Sakhis with the external world stimulated their aspirations to grow further and influenced choices made by them. A Sakhi-turned-master-trainer mentioned mobility adding to her confidence in being independent.

*“Now I can travel very far for work, I don’t feel afraid even if I am alone. Feel like my home wherever I go”.*

Another Sakhi discussed the value she found in interacting with experts and external contacts. Interestingly, these aspirations are not limited to herself or her family. A Sakhi mentioned,

*“I feel my village should always remain good and healthy, and zero mortality is my aim”.*

## Discussion

Based on the experiences shared by Sakhis about the process of their participation and self –growth through this process, an empowerment framework seems to be emerging. The process appeared to have begun with personal changes at an intrinsic level, which over the period of time expanded to the Sakhis’s standing in the society as an empowered woman involved in managing affairs of the community. The components of this process are described below sequentially, however, they are likely to have occurred parallel to each other with substantial overlaps.

### Evolution of a new self-identity

Sakhis experienced an emergence of a new identity for themselves which is clearly highlighted from an example. As a Sakhis mentioned, *“I might have remained just like that, uneducated and unhappy about not utilising my potential, if I had not been part of this program”.* Sakhis appeared to have learned to overcome their fears by confronting the fear and continuing with their work. As expressed by a Sakhi, *“I feel when we do something, initially we are scared but later the fear just withers*”. Over time, Sakhis were able to replace their initial fears with larger dreams for their own community’s development.

### Financial independence

Sakhis earned honorarium for their work. The earning, though not very substantial, provided Sakhis with respect and confidence which was highlighted in the example shared by a Sakhi.

*“I have two children and both are studying. One is pursuing BSc and other one is in 11* ^*th*^ *. When it came to paying their fees I used my earned money and took a decision to pay it off. My dream today is to ensure that my children are well educated”.*

It was evident in most cases that Sakhi’s earning became an additional support for the family and in most cases it was utilised for domestic needs and education of their children. Sakhis shared that, with access to finance, their involvement in household financial decisions improved. The finding corroborates with another study conducted by ACF which suggested the access to finance through self-earning regardless of the magnitude develops a sense of confidence among the women working as veterinary health workers [17].

### The growing circle of impact

The journey of Sakhis began with influencing family members for their consent to participate in the program. Non-cooperation and lack of support at family level in certain cases were replaced with acceptance and trust. A Sakhi who faced resistance initially mentioned, *“The decision benefited me a lot and my husband too turned supportive seeing the work I did”.* Another Sakhi shared, *“Now our family also doesn’t disallow us. In fact they say they are very proud of our work and earning money is inconsequential”.* In other words, as Sakhis started acquiring financial independence, they started getting a say in their family affairs and their role as Sakhi was accepted.

### Broadened development intercessions

Sakhis started being included in consultations related to other development agenda at the village level. The communities started seeking Sakhis to represent their interests by being on village level development committees such as education or sanitation committees. This elevated their status in the society. As a Sakhi expressed, *“Yes, I am respected today. If a meeting is held, they invite me in it. Whenever anew committee is formed, they always offer a post to me”.*

### Self-development to empowerment

The major learning as perceived by Sakhis in this case has specifically been in terms of improved knowledge, skills and attitudes. These aspects appeared to be interdependent and having a mutually fostering effect. The earned knowledge and learnt skills promoted positive attitude in the Sakhis, which in turn helped them take calculated risks and informed decisions while addressing difficult situations. The experience of successfully addressing difficult situations further accentuated the Sakhis’ self-image, increased their self-esteem, through vicarious learning and also strengthened their knowledge and skills. This ongoing process appeared to have triggered the process of empowerment in which the Sakhis’ growth was not limited to the personal level, but also at the societal level. Some Sakhis grew to become ASHAs or their supervisors and master trainers, others pursued education at secondary level and some also started working as CHW on other development projects. In other words, the Sakhis developed aspirations to perform effectively in future and not be without any constructive work. A Sakhi put this very simply and clearly – *“After being a Sakhi, I got habituated to being occupied. I am eager to work more in whatever way it may be”.*

## Conclusion

Sakhis, the key actors in the program, experienced a significant transformation in terms of improved ability in decision making which helped them to evolve their own identity. Sakhis place significant importance to creation of their identity. The elevated status for Sakhis in their community and an emergence of leadership reflected in their involvement in domain areas apart from health signifies empowerment.

Women, an imperative segment of the society, have often been denied a fair role in the process of development, especially in underprivileged areas. The program played an instrumental role in encouraging local women to participate and contribute in working for their communities’ health and development. Further, it paved a path for gender integration in development at a local level by providing opportunities for women to enhance and use their knowledge and skills for the development of their own community.

## Abbreviations

ACF: Ambuja Cement Foundation
ANC: Antenatal Care
CHW: Community-based Health Workers
CSR: Corporate Social Responsibility
FGD: Focussed Group Discussion
HBNC: Home Based Newborn Care
IFAD: International Fund for Agricultural Development
NGO: Non-Governmental Organization
PNC: Post-Natal Care
PSS: Pashu Swasthya Sevika
PTA: Parent-Teacher Association
UNICEF: United Nations International Children’s Emergency Fund
